# Exposure to advanced therapies and risk of surgery in Crohn’s disease

**DOI:** 10.1007/s00464-025-11919-7

**Published:** 2025-07-07

**Authors:** Marc M. Mankarious, Kara Dijoseph, Alicia C. Greene, Eric W. Schaefer, Kofi Clarke, Michael J. Deutsch, Jeffrey S. Scow, Afif N. Kulaylat, Audrey S. Kulaylat

**Affiliations:** 1 Department of Surgery, Pennsylvania State University, College of Medicine, Hershey, PA USA; 2 Division of Gastroenterology & Hepatology, Department of Medicine, Pennsylvania State University, Hershey, PA USA; 3 Department of Public Health Sciences, Penn State College of Medicine, Hershey, PA USA; 4 Division of Colon and Rectal Surgery, Department of Surgery, Pennsylvania State University, Hershey, PA USA

**Keywords:** Biologics, Crohn’s disease, Surgical resection, MarketScan, Postoperative complications

## Abstract

**Background & aims:**

While advancements in therapeutic options for inflammatory bowel disease reduced rates of surgical resection, some patients still require surgery despite multiple lines of medical therapies. This study investigates the relationship between the number, class, and progression rate of different advanced therapies (AT) and risk of surgical resection and postoperative complications in patients with Crohn’s Disease (CD).

**Methods:**

This study is a retrospective cohort study utilizing the MarketScan database, including adult patients with CD on AT from 2005 to 2020. The number of AT, class of AT, and comorbidities were assessed for all patients. The primary endpoint was surgical resection. A time-varying Cox proportional hazards regression model was used to assess risk of surgical resection. Logistic regression was used to assess secondary outcomes, including postoperative complications, readmissions, and emergency department (ED) visits.

**Results:**

The sample included 15,547 patients of whom 10.6% required surgical resection at some point. Use of anti-integrin therapy as first line was associated with higher risk of surgery compared to TNF-alpha inhibitors (Hazard ratio [HR] 1.39, *p* < 0.05). Utilization of 2, 3, or ≥ 4 AT was associated with increased surgery risk (HRs of 2.80, 4.54, and 7.82, respectively, *p* < 0.001). The number and class of ATs were not associated with postoperative complications, readmissions, or ED visits.

**Conclusion:**

Use of first-line anti-integrin was associated with higher risk of surgery and thus our study supports the use of TNF-alpha inhibitors as the first-line therapy in treatment-naïve patients. Nevertheless, multidisciplinary care is crucial in identifying and counseling patients at risk of surgery.

**Supplementary Information:**

The online version contains supplementary material available at 10.1007/s00464-025-11919-7.

The global prevalence of inflammatory bowel disease (IBD) has risen by 47.5% over the last 30 years [1]. Although IBD is associated with significant morbidity and reduced quality of life, recent advancements in the development of medical therapies have greatly improved the care and management of patients with IBD [2]. Specifically, multiple newer biologics and small molecules targeting various immunological pathways are now essential in treating patients with IBD [3–6]. Accordingly, there is an overall reduction in rates of surgical resection for patients with IBD over the past six decades [2, 7].

Despite this progress, some patients with severe diseases or associated complications still require surgical intervention. In patients with Crohn’s disease (CD), the overall 1-, 5-, and 10-year risk of surgery is approximately 19%, 28%, and 40%, respectively, based on a systematic review of population-based cohorts [8]. For patients with increased risk of surgical resection, variables associated with the increased risk of surgery include age at diagnosis, disease phenotype, and severity [9]. However, there is no clear understanding if and how the effect of the number, type, and rates of cycling through different advanced therapies modulate the risk of surgery in patients with CD.

We sought to evaluate the relationship between class, number, and rate of progression through various advanced therapies in CD patients and the impact on the likelihood of surgical resection and risk of postoperative complications.

We hypothesized that the use of an increasing number of advanced therapies (AT), defined by various targeted biologics and small molecules, would be associated with an increased risk of surgery without an increase in the risk of postoperative complications.

## Methods

### Study design, setting, and data sources

This is a retrospective cohort study using the MarketScan Commercial Claims and Encounters (*MarketScan, Merative*) database from 2005 to 2020. The database contains reimbursed claims within the USA from > 130 payers for > 56 million employees, dependents, and retirees covered annually under private insurance plans. No Medicaid or Medicare data are included. Patients are included for the duration of their enrollment in a given insurance plan, and the database includes inpatient claims, outpatient visits, emergency department visits, and pharmaceutical claims. The Human Subjects Protection Office at our institution determined that this project does not meet the definition of human subject research, and Institutional Review Board (IRB) review and approval were not required (Study ID: 00021077).

### Participants

We identified ATs using inpatient and outpatient claims for injections and pharmaceutical claims for prescriptions. Specifically, the Current Procedural Terminology (CPT) codes were used to identify injections of the following ATs: Infliximab (J1745), Adalimumab (J0135), Certolizumab (J0717), Vedolizumab (J3380), Golimumab (J1602), and Ustekinumab (C9487, Q9989, J3358). Pharmaceutical claims were queried for the following AT prescriptions: Infliximab, Adalimumab, Certolizumab Pegol, Vedolizumab, Golimumab, Ustekinumab, Risankizumab, and Ozanimod. The earliest claim date for an AT found in the database was used as the baseline (index date) in the analysis.

We included adult patients (≥ 18 years old) with at least 1 year of continuous enrollment in an insurance plan (with drug claims captured) prior to the index date. We used this 1 year look back to reduce likelihood that an AT was used prior to index date with another insurance provider. Patients with a diagnosis of CD in the 1 year prior to the index date were included using the following International Classification of Diseases, 9th and 10th (ICD-9/10) codes: 555.0–555.2, 555.9, K50.0x, K50.1x, K50.8x, and K50.9x. Patients were also required to have at least 30 days of continuous enrollment in an insurance plan after the index date.

Patients with a diagnosis of ulcerative colitis or other autoimmune conditions (rheumatoid arthritis, psoriatic arthritis, ankylosing spondylitis, plaque psoriasis, juvenile idiopathic psoriasis, hidradenitis suppurativa, uveitis, and active non-radiographic axial spondylitis) in 1 year prior to the index date were excluded. The reason for the exclusion is that the ATs we studied are often prescribed for these conditions. A detailed list of ICD codes used for these conditions is included in Supplemental Table S1. Patients with stricturing CD within 1 year prior to index date were also excluded (ICD-9: 560.8, 560.89, 560.9; ICD-10: K50.012, K50.112, K50.812, K50.912) as different treatment regimens are often recommended for this group of patients.

### Variables

#### Follow-up and ATs used

Follow-up time was calculated from the index date until the end date of continuous enrollment. The number of unique ATs and the timing of those ATs were determined from claims during the follow-up period. For example, if a patient had a claim for infliximab on the index date and a claim for adalimumab 200 days later, then the patient was considered to have 1 AT for the first 199 days and then 2 ATs starting on day 200.

ATs were also categorized based on mechanism of action: anti-TNF (infliximab, adalimumab, certolizumab, golimumab), IL inhibitors (Ustekinumab, Risankizumab), and integrin binders (vedolizumab).

#### Comorbidities

All inpatient and outpatient claims that occurred within 1 year prior to the index date were searched for claims with specific diagnosis codes [10, 11], and the Quan modification of the Charlson Comorbidity Index (CCI), which is based on 17 comorbidities, was subsequently calculated [10–12].

#### Outcomes

The primary endpoint was the time of first surgery involving bowel resection. We used the following CPT codes to identify these surgeries: small bowel resection and ileostomies (44110, 44111, 44120, 44121, 44125, 44130, 44202, 44203, 44205, 44227, 44316, 44602, 44603, 44615, 44640, 44650, 44660, 44661, and 45136), fistula repair (45804, 45820, 45825, 57305, 57307), subtotal colectomy or total abdominal colectomy (44140, 44141, 44143, 44144, 44145, 44146, 44147, 44150, 44151, 44152, 44153, 44160, 44204, 44206, 44207, 44208, and 44210), a total proctocolectomy (44155, 44156, 44212, 44157, 44158, and 44211), ostomy-related procedures (44187, 44188, 44310, and 44320), or proctectomy (45110, 45111, 45112, 45114, 45116, 45123, 45160, and 45395).

Postoperative complications were calculated from the date of surgery to 30-day post-discharge. Using Loftus et al. criteria, complications included fistula, abscess, stricture, sepsis/pneumonia/bacteremia, wound-related complications, anal/rectal repair or manipulation, return to the operating room for lysis of adhesions, and revision of ileostomy [13] (see Supplemental Table S2 for a list of complications with corresponding CPT and ICD-9/10 codes). Readmissions were defined as any inpatient claim within 30 days after discharge from surgery. Emergency department visits were determined from the service sub-category code in MarketScan, with a code having the last 2 digits of ‘20’ indicating an ED visit.

Use of steroids (prednisone, hydrocortisone, methylprednisolone) and immunomodulators (azathioprine, cyclosporine, and mercaptopurine) within 30 days prior to surgery was determined from inpatient, outpatient, and pharmaceutical claims. See Supplemental Table S3 for a list of generic names and HCPCS codes used to define each drug.

### Statistical methods

#### Landmark analysis

We conducted a landmark analysis using landmarks of 6, 12, 18, and 24 months after the index date to investigate the relationship between the number of ATs and surgery. For each landmark time, we focused on the subset of patients who had not yet had surgery and were still at risk for surgery, excluding those who had already had surgery or who had continuous enrollment cease (censored) prior to the landmark. The number of ATs was calculated from the index date up to the landmark time. The Kaplan–Meier method was used to estimate cumulative incidence curves by the number of ATs [14]. The log-rank test was used to test for differences among groups [15].

#### Time-varying analysis

A Cox model was fitted using the number of ATs as a time-varying covariate [16, 17]. The Cox model included the number of ATs (time-varying covariate) over time, age at index date, sex, year of index date, comorbidity index, and class of first AT received. Age, year, and comorbidity index were modeled linearly, which was appropriate based on spline fits and other graphical methods. All other variables were categorical and used reference coding. Hazard ratios (HRs) and corresponding 95% confidence intervals (CIs) were reported from the models. Schoenfeld residuals were used to verify that non-proportionality assumptions were appropriate [18].

#### Postoperative complications

The associations between the number of ATs prior to surgery and postoperative complications, ED visits, and readmissions were examined using logistic regression for the subset of patients who received surgery. The logistic regression models contained the following variables: number of AT, class of first AT received, age at surgery, sex, year of surgery, comorbidity index, steroid use prior to surgery, immunomodulator use prior to surgery, and the time from the index date until surgery. Age at surgery, year of surgery, comorbidity index, and time from the index date were all modeled using linear assumptions, which were appropriate based on spline fits. All other variables were modeled using reference categories. We reported odds ratios (ORs) and corresponding 95% CIs from these models.

## Results

### Participants and descriptive data

A total of 15,547 adult patients with CD were identified who met all inclusion and exclusion criteria. The median age was 38 (inter-quartile range [IQR] 27–50) (Table 1). Most patients had a comorbidity index of 0 (78.9%). Median follow-up time was 1.9 years (IQR: 0.8–3.8), with 10.6% of patients requiring surgical resection at some point during the follow-up period.

**Table 1:** Patient characteristics for all patients

*N*	15,547
Median age at index date (IQR)	38 (27–50)
Gender	
Male	7146 (46%)
Female	8401 (54%)
Comorbidity index	
0	12,273 (78.9%)
1	1575 (10.1%)
2	1251 (8%)
3	259 (1.7%)
≥ 4	189 (1.2%)
Median follow-up time, years (IQR)	1.9 (0.8–3.8)
Number of ATs	
1	12,607 (81.1%)
2	2347 (15.1%)
3	503 (3.2%)
4	80 (0.5%)
5	10 (0.1%)
First AT received (class)	
Anti-TNF	14,419 (92.7%)
Integrin binder	662 (4.3%)
IL inhibitor	466 (3%)
First AT received	
Adalimumab	8063 (51.9%)
Infliximab	5577 (35.9%)
Certolizumab	771 (5%)
Vedolizumab	662 (4.3%)
Golimumab	8 (0.1%)
Ustekinumab	466 (3%)
Surgery	
No	13,896 (89.4%)
Yes	1651 (10.6%)

The majority of patients (81.1%) did not switch AT during the follow-up period. Anti-TNF were used as the first-line AT in 92.7% of patients with Adalimumab (51.9%) and Infliximab (35.9%) being the most commonly used medications. Anti-integrins were used as first line AT in 4.3% of patients and IL inhibitors were used as first line in 3.0% of patients. A total of 19,180 unique ATs were used for these patients at some point during follow-up with 87% of these drugs being anti-TNFs.

### Landmark analysis

**Fig. 1:**
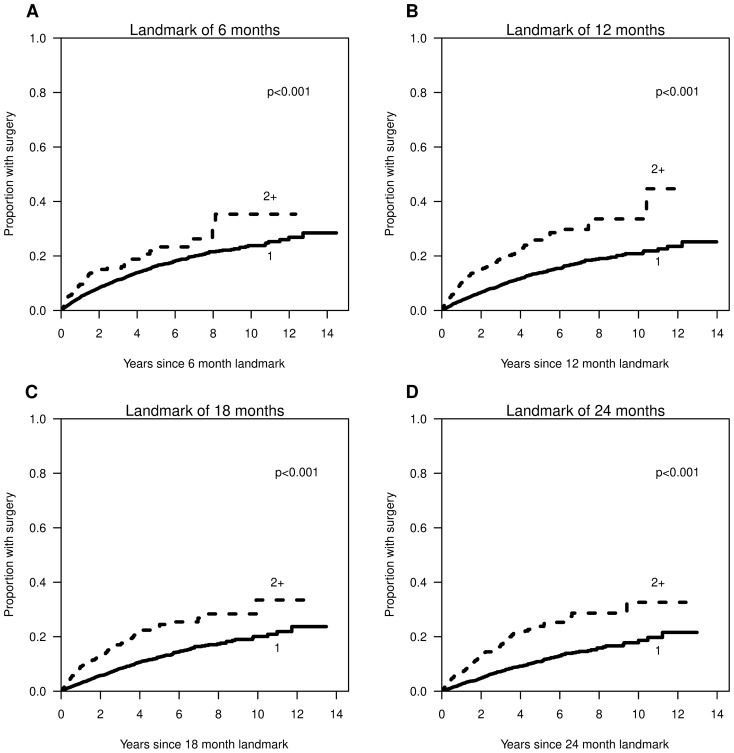
Cumulative incidence of surgical resection by number of advanced therapies (AT) received at landmarks of 6 (**A**); 12 (**B**); 18 (**C**); and 24 (**D**) months

For landmarks of 6, 12, 18, and 24 months, (Table 2), use of ≥ 2 ATs prior to landmark date was associated with higher cumulative incidence of surgical resection (Fig. 1A–D). Estimated HRs (95% CIs) from Cox regression models for ≥ 2 ATs versus 1 AT were 1.65 (1.31–2.07), 2.23 (1.88–2.65), 2.23 (1.86–2.68), and 2.48 (2.04–3.01) for 6, 12, 18, and 24 months, respectively.

**Table 2:** Sample sizes for landmark analyses

	Landmark at 6 months	Landmark at 12 months	Landmark at 18 months	Landmark at 24 months
Variable				
Number of patients included	12,944 (83.3%)	10,193 (65.6%)	8342 (53.7%)	6671 (42.9%)
Number of patients excluded	2603 (16.7%)	5354 (34.4%)	7205 (46.3%)	8876 (57.1%)
Reason for exclusion				
Surgery prior to landmark	478 (18.4%)	805 (15.0%)	1018 (14.1%)	1176 (13.2%)
Censored prior to landmark	2125 (81.6%)	4549 (85.0%)	6187 (85.9%))	7700 (86.8%)
Number of AT prior to landmark				
1	12,338 (95.3%)	9112 (89.4%)	7136 (85.5%)	5484 (82.2%)
2	603 (4.7%)	1031 (10.1%)	1099 (13.2%)	1038 (15.6%)
3	3 (0%)	49 (0.5%)	104 (1.2%)	145 (2.2%)
4		1 (0%)	3 (0%)	3 (0%)
5				1 (0%)

### Time-varying analysis

In the multivariable, time-varying Cox regression model, patients with 2, 3, and ≥ 4 AT (relative to 1 AT) had increasing hazards for surgical resection with HRs (95% CIs) of 2.80 (2.46–3.18), 4.54 (3.53–5.86), and 7.82 (4.53–13.5), respectively. The HRs were reasonably constant over time, as tests for non-proportionality over time were non-significant for the number of AT used. The use of anti-integrins as the first-line therapy for CD was associated with increased higher HR (1.39, 95% CI 1.03–1.87) of surgery compared to patients in whom anti-TNF were the first-line therapy (Table 3). Increasing age at index date had lower HR for surgery with 1-year increase (HR 0.92, 95% CI 0.88–0.95).

**Table 3:** Estimated HRs from fitted cox regression model using the number of ATs as a time-varying covariate

Parameter	HR (95% CI)	*p* value
First AT received		
Anti-TNF (ref)	1	
IL inhibitor	1.26 (0.84–1.89)	0.27
Integrin binder	1.39 (1.03–1.87)	0.031
Number of AT		
1 (ref)	1	
2	2.80 (2.46–3.18)	< 0.001
3	4.54 (3.53–5.86)	< 0.001
4 or more	7.82 (4.53–13.5)	< 0.001
Age at index date, 1-year increase	0.92 (0.88–0.95)	< 0.001
Male sex (vs. female)	1.01 (0.92–1.12)	0.77
Year of index date, 1-year increase	0.93 (0.92–0.94)	< 0.001
Comorbidity index, 1-point increase	0.97 (0.91–1.03)	0.32

#### Postoperative complications

A total of 1651 patients had surgical resection within the follow-up period, of whom 22.3% used steroids and 11.9% used immunomodulators within 30 days prior to surgery (Table 4). The median number of days from index date of starting first AT to surgical resection was 377 (IQR 151–832), with the majority of patients using 1 unique AT (73.7%) prior to operation.

**Table 4:** Baseline characteristics and postoperative complications for patients undergoing surgery

*N*	1651
Median age at surgery (IQR)	39 (28–50)
Median days from index date to surgery	377 (151–832)
Sex	
Male	746 (45.2%)
Female	905 (54.8%)
Comorbidity index	
0	1335 (80.9%)
1	160 (9.7%)
2	123 (7.5%)
3	23 (1.4%)
≥ 4	10 (0.6%)
Steroids within 30 days prior to surgery	368 (22.3%)
Immunomodulator within 30 days prior to surgery	196 (11.9%)
Number of unique AT prior to surgery	
1	1217 (73.7%)
2	348 (21.1%)
3	72 (4.4%)
4	13 (0.8%)
5	1 (0.1%)
First AT received	
Anti-TNF	1578 (95.6%)
IL inhibitor	25 (1.5%)
Integrin binder	48 (2.9%)
Fistula	451 (27.3%)
Abscess	372 (22.5%)
Stricture	110 (6.7%)
Sepsis–pneumonia–bacteremia	204 (12.4%)
Wound dehiscence	66 (4%)
Anal/rectal repair or manipulation	56 (3.4%)
Lysis of adhesions	26 (1.6%)
Revision of ileostomy	20 (1.2%)
Any complication (above list)	855 (51.8%)
ED visit	319 (19.3%)
Readmission	254 (15.4%)

Rates of any complication, ED visits, and readmissions within 30-day post-surgery were 51.8%, 19.3%, and 15.4%, respectively. Incidence rates for specific complications (fistula, abscess, stricture, sepsis, wound dehiscence, anal/rectal repair or manipulation, lysis of adhesions, and revision of ileostomy) are listed in Table 4. From multivariable logistic regression, number of ATs used and first class of AT were not associated with any complication, ED visits, or readmissions (Table 5). Factors associated with increased likelihood of any postoperative complications included male sex (OR 1.36, 95% CI 1.12–1.66) and immunomodulator use within 30 days prior to surgery (OR 1.37, 95% CI 1.01–1.85).

**Table 5:** Estimated ORs from fitted logistic regression model for any complication, ED visits, and readmissions

Parameter	Any Complication		ED Visit		Readmissions	
	OR (95% CI)	*p* value	OR (95% CI)	*p* value	OR (95% CI)	*p* value
Number of AT						
1 (ref)	1		1		1	
2	0.92 (0.71–1.18)	0.51	1.07 (0.78–1.47)	0.68	1.39 (0.99–1.94)	0.06
3 or more	1.36 (0.84–2.21)	0.21	1.75 (1.03–2.98)	0.04	1.67 (0.92–3.04)	0.09
Age at surgery, 10-year increase	0.97 (0.90–1.05)	0.43	1.00 (0.91–1.10)	0.98	1.03 (0.92–1.14)	0.62
Male sex (vs. female)	1.36 (1.12–1.66)	0.002	0.87 (0.68–1.11)	0.26	1.04 (0.79–1.36)	0.79
Year of surgery, 1-year increase	0.99 (0.96–1.02)	0.66	0.99 (0.95–1.02)	0.49	0.94 (0.91–0.98)	0.007
Comorbidity index, 1-point increase	1.07 (0.94–1.23)	0.32	1.11 (0.94–1.31)	0.2	1.01 (0.84–1.22)	0.91
Steroid within 30 days prior to surgery	1.12 (0.88–1.41)	0.35	1.08 (0.81–1.45)	0.59	1.23 (0.90–1.68)	0.19
Immunomodulator within 30 days prior to surgery	1.37 (1.01–1.86)	0.045	1.17 (0.81–1.69)	0.42	1.18 (0.79–1.76)	0.42
Time from index date to surgery, 1-year increase	0.99 (0.93–1.06)	0.87	1.06 (0.98–1.14)	0.15	1.07 (0.99–1.16)	0.09
First AT received						
Anti-TNF (ref)	1		1		1	
IL inhibitor	1.09 (0.48–2.45)	0.84	0.64 (0.19–2.21)	0.48	1.16 (0.33–4.04)	0.81
Integrin binder	1.17 (0.64–2.13)	0.61	1.42 (0.70–2.90)	0.34	2.04 (0.96–4.31)	0.06

## Discussion

Our study describes the risk of surgery in CD patients treated with AT and the associated risk based on number of advanced therapies used. In addition, our study showed an increased risk of surgical resection in patients who used anti-integrin molecules as the first-line therapy compared to using anti-TNF. The American Gastrointestinal Association (AGA) recommends the use of anti-TNF over no treatment for induction and maintenance of remission while also acknowledging that the use of vedolizumab is also reasonable with low evidence for induction and moderate evidence for maintenance [3]. The European Crohn’s and Colitis Organization (ECCO) also recommends the use of anti-TNF as the first-line therapy in moderate-to-severe Crohn’s disease while reserving the use of vedolizumab in patients with inadequate response to anti-TNF therapy [4]. On the other hand, other guidelines from the British Society of Gastroenterology (BSG) and the Italian Group for the Study of Inflammatory Bowel Disease (IG-IBD) do not differentiate which drug class should be utilized first in AT naive patients [5, 6]. This is likely due to the limited evidence regarding the risks and benefits of using anti-TNF versus vedolizumab as the first-line therapy. A network meta-analysis of 15 randomized control trials incorporating 2931 patients noted that in biologic-naïve patients, there was low confidence estimates supporting the use of infliximab or vedolizumab as the first-line therapy (OR 3.76 [95% CI 1.01, 14.03]) [19]. Accordingly, our data support the use of anti-TNF as the first-line regimen in AT-naïve patients as the use of vedolizumab as the first-line therapy for CD is associated with higher hazard ratio for surgery compared to anti-TNF medications. This appears to be in line with clinical practice in the USA as our data suggest that anti-TNF is a first-line AT in 92.7% of patients, while integrin binders are used in 4.3% of patients.

Although ATs continue to offer an excellent treatment option for patients with CD, our study highlights the importance of multidisciplinary care with early involvement of surgical teams in patients with CD. Previous work has shown that, within certain subsets of patients, early surgery might be more advantageous to ongoing AT therapy. In a Markov model of patients with limited non-stricturing ileocecal CD, patients with early surgery had lower mean costs ($29,457 vs $50,382) and higher quality-adjusted life years (QALYs) (6.24 vs. 5.81) compared to patients with biologic therapy [20]. Similarly, studies of the European and Asian cohorts noted lower utilization of biologics with early surgery in particular cohorts or patients with CD [21–23]. Within our cohort, although we are unable to identify different phenotypes of CD, we note that the hazard rate of surgical resection approximately doubles with each new AT used for patients treated with 2 or more ATs over their disease course. This points to a subset of patients in whom surgical resection is warranted at an earlier timepoint depending on disease progression.

When patients with CD who utilized multiple ATs over their disease course require surgery, our study shows that the number of ATs utilized preoperatively is not associated with an increased risk of postoperative complications. While several retrospective studies and meta-analyses showed mixed data regarding the risk of postoperative complications and preoperative use of ATs, the PUCCINI trail, a randomized control trial of patients with IBD from 17 different centers, did not find an association between anti-TNF exposure and detectable levels preoperatively and risk of infectious postoperative complications [11, 24–27]. Notably, however, none of these studies examined the number of ATs used preoperatively, an important element that our study contributes to the growing body of literature that ATs are not associated with increased postoperative complications.

Interestingly, in our cohort, utilization of anti-integrins as the first-line therapy was associated with increased risk of surgical resection. This could be related to several factors related to patient selection or duration to induction. In a real-world retrospective review of biologic-naive CD patients started on vedolizumab vs anti-TNF therapies, baseline characteristics of the cohorts showed patients started on vedolizumab were older (median age 51.7 vs. 39.7, *p* < 0.01) and had longer disease duration ≥ 5 years (53.4% vs. 35.9%, *p* < 0.01) [28]. While outcomes were equivalent between the groups after propensity score matching, this points to a possible baseline selection bias by providers that may impact the outcomes seen in this study. In another study of biologic-naive patients, vedolizumab initially resulted in significantly lower induction rates compared to anti-TNF (56.3% vs 73.9%, *p* < 0.05). However, at the 2-year mark, vedolizumab demonstrated significantly higher clinical remission rates compared to anti-TNF (74.2% vs 44.7%; *p* < 0.05) [29]. Therefore, it is possible within our cohort that longer duration to induction led to a medication switch or progression to surgical intervention.

This study has several limitations. Perhaps the greatest limitation is the shorter median follow-up of 1.9 years. This is unfortunately inherent to an insurance-claims database and could not be bypassed methodologically. However, while that may mean that rates of surgical resection and risk of surgery could be different with longer follow-up, data still provide prognostic information within the first 2 years of starting the first AT. As with database studies, our study is limited by the absence of granular information related to disease characteristics, such as disease severity, endoscopic scoring systems, drug levels, pathological findings, presence of dysplasia, and laboratory values. In addition, data regarding quality of life of patients on different numbers of ATs could not be examined. Furthermore, our study was limited to patients under the age of 65 with private insurance, and the proportion of patients who underwent an AT switch secondary to side effect intolerance versus refractory disease is unknown. We attempted to exclude patients who had been on previous ATs by ensuring that there were no claims for another AT for 1 year prior to our “index date.” However, it is possible that some patients had received other ATs while enrolled in a separate insurance plan and then were off therapy for > 1 year prior to receiving an AT under their new plan; this would mean that the “first” AT may not, in special circumstances, be the patient’s first AT, although we assume that this occurrence would be rare. We recognize that anti-integrin molecules may have been used as the first-line therapy in patients with stricturing disease which may be a risk factor for surgical resection. Therefore, to eliminate this potential confounding, patients with diagnoses indicating stricturing disease prior to first AT were excluded.

## Conclusion

While the use of ATs is associated with overall lower rates of surgery for patients with CD, patients who cycle through ≥ 2 ATs are at higher risk of requiring surgical resection. Additionally, patients utilizing vedolizumab as the first-line therapy are at increased risk of requiring surgical resection compared to patients utilizing anti-TNF as first line. Utilization of multiple ATs and class of first AT utilized are not associated with risk of postoperative complications, ED visits, or readmissions. These findings highlight the importance of counseling patients on the risk of surgery as they progress through various drugs. Accordingly, this should prompt potentially the earlier involvement of surgical teams in the care of patients cycling through multiple drugs.

## Supplementary Information

Below is the link to the electronic supplementary material.

Supplementary file1 (DOCX 18 KB)

## Data Availability

Data available by request to corresponding author. Availability subject to contractual constraints with MarketScan team.

## Abbreviations

CD: Crohn’s disease
AT: Advanced therapies
IBD: Inflammatory bowel disease
CPT: Current procedural terminology
ICD-9/10: International Classification of Diseases 9th and 10th edition
anti-TNF: TNF-alpha inhibitors
ED: Emergency department
