# Supplementary material for: Exposure to advanced therapies and risk of surgery in Crohn’s disease
Source: Surg Endosc. 2025 Jul 7;39(8):5367–75. doi: 10.1007/s00464-025-11919-7 (PMC12287203; doi:10.1007/s00464-025-11919-7)
Supplement: Supplementary file 1 — Supplementary file1 (DOCX 18 KB) [file 464_2025_11919_MOESM1_ESM.docx]

**Supplemental Tables/Figures:**

**S1: ICD Codes for Excluded Conditions:**

| **Disease** | **ICD-9** | **ICD-10** |
| --- | --- | --- |
| Ulcerative Colitis | 556.0-556.6, 556.8, 556.9 | K51.X |
| Rheumatoid arthritis | 714.0, 714.1, 714.2 | M05.X, M06.X |
| Psoriatic Arthritis | 696.0 | L40.5X |
| Ankylosing Spondylitis | 720.0 | M45.X |
| Plaque Psoriasis | 696.1 | L40.0 |
| Juvenile idiopathic arthritis | 714.3X, 714.30, 714.31, 714.32, 714.33 | M08.X |
| hidradenitis suppurativa | 705.83 | L73.2 |
| Uveitis | 360.11, 360.12 | H20.01, H20.02, H20.04, H20.1 |
| Active non-radiographic axial spondyloarthritis | 720.X | M45.AX |

| **Table S2: CPT and ICD-9/10 diagnosis codes used to define complications (Modified from Loftus et. al.)** | |
| --- | --- |
| **Complication** | **Codes** |
| Fistula | CPT: 44640, 44650, 44660, 44661, 45800, 45805, 45820, 45825, 46020, 46030, 46258, 46262, 46270, 46275, 46280, 46285, 46288, 46706, 46710, 46712, 46715, 46716, 46740, 46742, 57300, 57305, 57307, 57308  ICD-9 diagnosis: 537.4, 565.1, 569.69, 569.81, 575.5, 593.82, 596.1, 596.2, 599.1, 608.89, 619.0, 619.1, 619.2, 998.6  ICD-10 diagnosis: K31.6, K60.3, K60.4, K60.5, K94.09, K94.19, K63.2, K82.3, N28.89, N32.1, N32.2, N36.0, N50.8, N82.0, N82.4, N82.5, T81.83XA |
| Abscess | CPT: 10060, 10061, 10140, 10160, 10180, 20000, 45000, 45005, 45020, 46040, 46045, 46050, 46060, 49020, 49021, 49040, 49041, 49061, 49062  ICD-9 diagnosis: 540.1, 566, 567.2, 569.5, 569.61, 595.89, 597.0, 614.4, 682.2, 682.5, 682.9  ICD-10 diagnosis: K35.3, K61.0, K61.1, K61.3, K63.0, K65.1, K65.9, K94.02, K94.12, N30.80, N30.81, N34.0, N73.2, L02.210, L02.211, L02.212, L02.214, L02.215, L02.216, L02.219, L02.31, L02.91, K63.0 |
| Stricture | CPT: 44615, 45150, 45910, 46700  ICD-10 diagnosis: K62.4, K91.89, K91.30, K91.31, K91.32, K91.33 |
| Sepsis-pneumonia-bacteremia | ICD-9 diagnosis: 038.0, 038.10, 038.11, 038.19, 038.2, 038.3, 038.40, 038.41, 038.42, 038.43, 038.49, 038.8, 038.9, 480.0, 480.1, 480.2, 480.3, 480.8, 480.9, 481, 482.0, 482.1, 482.2, 482.3, 482.30, 482.31, 482.32, 482.39, 482.40, 482.41, 482.49, 482.81, 482.82, 482.83, 482.84, 482.89, 482.9, 483.0, 483.1, 483.8, 484.1, 484.3, 484.5, 484.6, 484.7, 484.8, 485, 486, 599.0, 790.7, 999.3  ICD-10 diagnosis: A40.x, A41.x, J12.x, J13.x, J14.x, J15.x, J16.x, J17.x, J18.x, R65.x, R78.81, T80.2, N39.0, T81.12, T81.44 |
| Wound debridement and dehiscence | CPT: 11000, 11001, 11004, 11005, 11006, 1008, 11010, 11011, 11012, 11040, 11041, 11042, 11043, 11044, 12020, 12021, 13160, 49900, 97602, 97597, 97598  ICD-9 diagnosis: 998.31, 998.32  ICD-10 diagnosis: T81.3, T81.41, T81.42 |
| Anal/rectal repair or manipulation | CPT: 44602, 44603, 44604, 44605, 44700, 44701, 44799, 45500, 45505, 45520, 45540, 45541, 45550, 45560, 45562, 45563, 45900, 45905, 45910, 45915 |
| Lyses of adhesions | CPT: 44005, 44180, 44200 |
| Revision of ileostomy | CPT: 44312, 44314, 44340, 44345, 44346 |

| **Table S3. Generic names and HCPCS codes used to define each drug** | | |
| --- | --- | --- |
|  | **Generic name** | **HCPCS** |
| **Steroids** |  |  |
| Prednisone | Prednisone | J7506 |
| Hydrocortisone | Hydrocortisone | J1700, J1710, J1720 |
| Methylprednisolone | Methylprednisolone | J1020, J1030, J1040, J2920, J2930, J7509 |
|  |  |  |
| **Immunomodulators** |  |  |
| Azathioprine | Azathioprine | J7500, J7501 |
|  |  |  |
| Cyclosporine | Cyclosporine | J7502, J7515, J7516 |
|  |  |  |
| 6-mercaptopurine | Mercaptopurine | S0108 |
